# Discovery and Confirmation of Ligand Binding Specificities of the *Schistosoma japonicum* Polarity Protein Scribble

**DOI:** 10.1371/journal.pntd.0002837

**Published:** 2014-05-01

**Authors:** Pengfei Cai, Yi Mu, Xianyu Piao, Nan Hou, Shuai Liu, Youhe Gao, Heng Wang, Qijun Chen

**Affiliations:** 1 MOH Key Laboratory of Systems Biology of Pathogens, Institute of Pathogen Biology, Chinese Academy of Medical Sciences & Peking Union Medical College, Beijing, The Peoples Republic of China; 2 National Key Laboratory of Medical Molecular Biology, Department of Physiology and Pathophysiology, School of Basic Medicine, Peking Union Medical College, Institute of Basic Medical Sciences, Chinese Academy of Medical Sciences, Beijing, The Peoples Republic of China; 3 Department of Microbiology and Parasitology, Institute of Basic Medical Sciences, Chinese Academy of Medical Sciences & School of Basic Medicine, Peking Union Medical College, Beijing, The Peoples Republic of China; 4 Key Laboratory of Zoonosis, Ministry of Education, Institute of Zoonosis, Jilin University, Changchun, The Peoples Republic of China; Institute of Tropical Medicine (NEKKEN), Japan

## Abstract

**Background:**

Schistosomiasis is a chronic debilitating parasitic disease that afflicts more than 200 million individuals worldwide. Long-term administration of chemotherapy with the single available drug, praziquantel, has led to growing concerns about drug resistance. The PSD-95/Dlg/ZO-1 (PDZ) domain is an important module found in many scaffolding proteins, which has been recognized as promising targets for the development of novel drugs. However, the parasite-derived PDZ domains and their associated functions are still largely unknown.

**Methodology/Principal Findings:**

The gene encoding the *Schistosoma japonicum* Scribble protein (SjScrib) was identified by homologous search with the *S. mansoni Scrib* sequence. By screening an arbitrary peptide library in yeast two-hybrid (Y2H) assays, we identified and confirmed the ligand binding specificity for each of the four PDZ domains of SjScrib. Both SjScrib-PDZ1 and SjScrib-PDZ3 recognize type I C-terminal PDZ-domain binding motifs (PBMs), which can be deduced as consensus sequences of -[Φ][x][E][TS][x][ILF] and -[x][RKx][E_TS_][T][WΦ][ILV], respectively. SjScrib-PDZ2 prefers stringent type II C-terminal PBMs, which significantly differs from that of its human ortholog. SjScrib-PDZ4 binds to typical II C-terminal PBMs with a consensus sequence -[x][F_W_][x][LI][x][LIV], in which the aromatic residue Phe is predominantly selected at position -4. The irregular and unconventional internal ligand binding specificities for the PDZ domains of SjScrib were confirmed by point mutations of the key amino acids within the ligand binding motifs. We also compared the differences in ligand specificities between SjScrib-PDZs and hScrib-PDZs, and explored the structural basis for the ligand binding properties of SjScrib-PDZs.

**Conclusions/Significance:**

In this study, we characterized and confirmed the ligand binding specificities of all four PDZ domains of SjScrib for the first time. We denoted the differential ligand binding specificities between SjScrib-PDZs and hScrib-PDZs as well as the structural basis for these properties. This work may provide a fundamental basis for the rational design of novel anti-schistosomal drugs.

## Author Summary

Schistosomiasis japonica remains a major public health problem in China and Southeast Asia. The long-term of treatments with the only available drug, praziquantel, has raised the concerns about drug resistance. Protein-protein interactions (PPIs), for highly discriminating specificities, are thought to be the innovative targets for a generation of new drugs. The PDZ domain is one of the most important modules for PPIs. A number of compounds screened based on binding specificities of PDZ domains have shown their potential therapeutic power in several disease models with less side effects. Although domain loss events are widespread in *S. japonicum*, a panel of PDZ domains is conserved in this species. So far, however, little is known about ligand binding specificities and the molecular functions of parasite-derived PDZ domain-containing proteins. In this study, by yeast two-hybrid screening of a random library, we confirmed the ligand binding properties of a multiple PDZ domain-containing protein Scribble of *S. japonicum* for the first time. Divergent ligand specificities between the homologous PDZ domains of *S. japonicum* and human Scribble orthologs were revealed. Internal motif recognition and irregular ligand interaction models for the SjScrib-PDZ domains were identified. These results provide an important basis for the rational discovery of anti-schistosomal drugs.

## Introduction

Schistosomiasis, caused by members of the genus *Schistosoma*, afflicts more than 200 million people living in the endemic areas of 77 countries worldwide, representing a major health and economic burden in tropical and developing nations [1]. The treatment of schistosomiasis is largely based on the long-term application of the single available drug, praziquantel (PZQ). However, concerns were raised about the problem of drug resistance [2], [3]. The advances of schistosome genomics have provided a valuable basis for dissecting the parasite biology and identifying novel drug targets against the parasite [4]–[6].

Protein-protein interactions (PPIs) mediate multiple biological processes and physiological functions in organisms, and offer a variety of opportunities for exploring potential drug targets [7]. The PSD-95/Dlg/ZO-1 (PDZ) domain is one of the most crucial modules for PPI, and is emergently recognized as a novel target of drug discovery [8]–[11]. PDZ domains consist of approximately 80–90 amino acids, that typically form a packed structure composed of six β-strands (β1-β6) and two α-helices (α1, α2) [12]. A hydrophobic pocket formed with the carboxylate-binding loop (β1-β2 loop), β2 strand and the α2 helix of the PDZ domain can accommodate canonical four to five amino acids at the C-termini of partner proteins [13]. The PDZ-domain binding motifs (PBMs) have been conventionally classified into four categories based on the extreme C-terminus: Class I, -[S/T]-*X*-Φ*; Class II, -*Φ*-*X*-Φ*; Class III, -[D/E/K/R]-*X*-Φ*; and Class IV, -*X*-*ψ*-[D/E]*; where *X* is any amino acid, *Φ* is a hydrophobic residue, *ψ* is an aromatic residue, and * represents the stop codon of the peptide [14]. Although most of PDZ domains prefer binding of the extreme C-termini of their target ligands, several of them can also recognize internal motifs of their partner proteins [15], [16].

Scribble is a member of the evolutionarily conserved LAP (LRR (leucine-rich repeats) and PDZ) protein family. The N-terminal LRR domain is necessary for targeting to the basolateral membrane of epithelial cells [17], [18], and can interact with the PH domain leucine-rich-repeat protein phosphatase 1 (PHLPP1) to negatively regulate Akt signalling [19], while the PDZ domains of Scribble mediate interactions with a number of partners, such as β-PIX (PAK-interacting exchange factor β) [20], Vangl2, a core planar cell polarity (PCP) protein [21]–[23], NOS1AP [24], zyxin [25], and ZO-2 [26], as well as proteins from pathogenic viruses [27]–[29]. Scribble has been implicated to be involved in establishing and maintaining membrane polarity in epithelia, neurons, and T cells [30]–[33], mediating PCP signalling [21], [34], [35], regulating cell migration [36], [37] and act as a tumor suppressor [38]–[41]. Also, it has been shown that Scribble plays an important role in clustering synaptic vesicles at developing synapses via interaction with β-catenin [42]. The binding specificity profiles for a number of PDZ domains of LAP proteins, including human Scribble (hScrib), have been investigated by screening phage display library, most of which were found to prefer typical I C-terminal PDZ binding motifs (PBMs) [43].

In this study, by yeast two-hybrid (Y2H) screening of a random peptide library and introducing point mutations, we revealed and confirmed the ligand binding specificities, including the canonical C-terminal and non-canonical internal binding specificities for all four PDZ domains of the *S. japonicum* cell polarity protein Scribble (SjScrib). The binding specificity profiles were compared with those of human Scrib ortholog. The potential ligands of SjScrib were further predicted based on the ligand binding specificities of the PDZ domains, which were validated in the Y2H system. This work will facilitate the identification of novel drug targets against schistosome infection.

## Methods

### Ethical statement

All procedures carried out on animals within this study were conducted following animal husbandry guidelines of the Chinese Academy of Medical Sciences and with permission from the Experimental Animal Committee of Chinese Academy of Medical Sciences with the Ethical Clearance Number IPB-2011-6.

### Parasites and animals

*Schistosoma japonicum*-infected *Oncomelania hupensis* were purchased from Jiangxi Institute of Parasitic Diseases, Nanchang, China. Cercariae were freshly shed from the infected snails. New Zealand White rabbits were percutaneously infected with ∼1,000 *S. japonicum* cercariae. Hepatic schistosomula and adult worms were recovered from the rabbits by hepatic-portal perfusion at 2 and 6 weeks post-infection (p.i.), respectively. Male and female adult worms were manually separated with the aid of a light microscope. Eggs were isolated from the liver tissues of the infected rabbits at 6 weeks p.i. by the sieving and enzymatic digestion method [44].

### Cloning of the *S. japonicum* Scribble gene

Two EST sequences (GenBank accession number AY809835 and AY815909), respectively encoding the N-terminal and C-terminal fragment of SjScrib, were identified and retrieved directly from NCBI using the *S. mansoni Scribble* cDNA sequence (GenBank accession number: XM_002581163) as a bait. Forward (5′-GGCTCATAATGTTCAAGTGTTTGCCAATTATAGG-3′) and reverse (5′-CCAGAAGTGCTCCCATATTGTGCGTCATCG-3′) primers were designed based on these EST sequences and used to amplify the full-length ORF of *SjScrib* from adult worm pair cDNA templates with high fidelity Phusion DNA polymerase (New England Biolabs, NEB, UK). The resulting PCR fragment was cloned into T-vector and sequenced. The amino acid sequence of SjScrib (GenBank accession number: AHC92618) was deduced from the obtained cDNA sequence (GenBank accession number: KF730248) for further bioinformatic analysis.

### Bioinformatical analysis of SjScrib

The amino acid sequences of the Scrib orthologs of *S. mansoni* (GenBank accession number: CCD76510), *Drosophila melanogaster* (GenBank accession number: NP_733154) and *Homo sapiens* (GenBank accession number: AAL38976) were retrieved from GenBank. The domain boundaries of SjScrib and hScrib were analyzed using the following public domain tools: the NCBI CDD (http://www.ncbi.nlm.nih.gov/cdd) [45], PFAM [46], and SMART [47]. Peptide sequences of all PDZ domains from SjScrib, SmScrib, and hScrib were aligned by ClustalX 2.0. The structure-based alignment of these PDZ domains was produced by ESPript [48]. The three-dimensional (3D) structures of SjScrib-PDZ1, SjScrib-PDZ2 and SjScrib-PDZ3 were predicted by the online service Phyre 2 (http://www.sbg.bio.ic.ac.uk/phyre2/html/page.cgi?id=index) [49]. The 3D structure of SjScrib-PDZ4 was predicted based on the NMR structures of hScrib-PDZ1 (PDB code 1X5Q), hScrib-PDZ2 (PDB code 1WHA), and hScrib-PDZ4 (PDB code 1UJU) using the program EasyModeller 4.0 [50]. The structural alignments of PDZ domains between SjScrib and hScrib were performed by using the Swiss PDB Viewer (SPDBV) [51]. The solution NMR structures of hScrib-PDZ1 (PDB code 1X5Q), hScrib-PDZ2 (PDB code 1WHA), and hScrib-PDZ4 (PDB code 1UJU) were used as templates for superposition of the predicted structures of SjScrib-PDZ1, SjScrib-PDZ2, and SjScrib-PDZ4, respectively. The superimposed structures were further refined with PyMOL Viewer program (DeLano Scientific, San Carlos, CA, http://www.pymol.org/). Free, open source Java software for visualizing ligand binding specificity profiles is available from http://baderlab.org/Software/LOLA.

### Transcriptional characterization of the *SjScrib* gene by quantitative RT-PCR

Total RNAs of *S. japonicum* at different developmental stages (cercariae, hepatic schistosomula, separated male and female adult worms, and eggs) were extracted using Trizol reagent (Invitrogen, CA, USA). The possible contaminating genomic DNA was completely removed from RNA samples with TURBO DNA-free kit (Ambion, CA, USA). RNA quantification and quality control was conducted by 1% agarose gel electrophoresis and the Nanodrop ND-1000 spectrophotometer (Nanodrop Technologies, Wilmington, DE). For each sample, 1 µg total RNA was reverse transcribed into first-strand cDNA using SuperScript III Reverse Transcriptase Kit (Invitrogen). The synthesis procedure was performed as follows: 25°C for 5 min, 50°C for 1 h, 70°C for 15 min. Each PCR reaction contained 12.5 µl of 2×Brilliant II SYBR Green QPCR Master Mix (Agilent, USA), 1 µl diluted cDNA (20×), 1 µl of the forward and reverse primer pair (Table S1), and 10.5 µl of sterile water. The PCR program included 40 cycles with denaturation at 95°C for 30 s, followed by annealing and extension at 60°C for 1 min. Quantification of the transcriptional level of the *SjScrib* gene was performed by normalizing against the *PSMD4* transcript (26S proteasome non-ATPase regulatory subunit 4, GenBank Accession Number: FN320595) [52], [53] and applying the comparative 2^−ΔΔCt^ method, according to the SDS 1.4 software.

### Construction of bait plasmids and Y2H screening of a random peptide library

Primer pairs were designed based on the boundaries of PDZ domains (shown in Table S1). The DNA fragments encoding PDZ domains of SjScrib were respectively amplified from the adult worm cDNA templates using Phusion DNA polymerase. The PCR was performed with an initial denaturation for 1 min at 98°C, followed by thirty cycles: 98°C for 5 s; 50°C for 30 s and 72°C for 15 s. The final extension was 5 min at 72°C. The PCR products were digested with *Eco*R I and *Bam*H I, and cloned into the *Eco*R I*/Bam*H I sites of the GAL4 BD vector, pBridge (Clontech).

Each GAL4 BD-fusion bait plasmid was transformed into the yeast strain CG1945 using the lithium acetate procedure. The transformants were grown on SD/-Trp plates and *lacZ* assays were performed to examine self-activation. The transformants were further spread on SD/-His/-Trp plates with different concentration of 3-amino-1,2,4-triazole for leakage test. A random peptide library (constructed with *Tsp*509I-digested human genomic DNA fragments) [54] was screened following the MATCHMAKER Two-Hybrid System protocol (Clontech). The transformants were screened on SD/-Trp/-His/-Leu plates with different concentration of 3-amino-1,2,4-triazole (2.5 mM for the SjScrib-PDZ1, SjScrib-PDZ2, and SjScrib-PDZ4 bait transformants, and 7.5 mM for the SjScrib-PDZ3 bait transformant) and further verified by the improved *LacZ* assays. After rescue, the potential positive plasmids were isolated and retransformed into the yeast strain CG1945 containing corresponding bait plasmid. Only the clones that were positive for all the reporter assays and confirmed by at least two independent tests were selected for specific interactions and sequenced [55].

### Confirmation of the potential binding site within irregular ligands

For each irregular ligand, a putative binding site was deduced from the pattern of regular C-terminal PDZ binding motifs. Point mutation was induced by PCR against key residues within the putative binding site. DNA fragments produced by self primer template PCR reactions or amplified from ligand plasmids were digested with *Eco*R I and *Bam*H I, and cloned into the GAL4 BD vector, pBridge. Each clone was subjected to sequencing to make sure that the mutation was correctly introduced. Each of the mutated plasmids was co-transformed with the corresponding PDZ domain bait plasmids into yeast strain CG1945, respectively. The interactions between the mutants and the corresponding PDZ domain were validated by Y2H assays as mentioned above.

### Prediction of native ligand candidates

The consensus C-terminal binding sequences for each SjScrib-PDZ domain were deduced from sequence alignments of positive clones from Y2H screening. The *S. japonicum* predicted peptide sequences were downloaded from SDSPB (http://lifecenter.sgst.cn/schistosoma/cn/schdownload.do). The Tailfit software [56] was used to search against the *S. japonicum* peptide dataset to retrieve the potential ligands whose carboxyl termini matched the consensus-binding sequences. These peptide sequences were then manually BLAST in NCBI to filter the truncated fragments lacking of the C-terminus. Further, the most promising candidates were selected based on biological information such as subcellular localization and potential molecular function, as well as the C-terminal homology between *S. japonicum* and *S. mansoni* orthologs [57].

### Confirmation of candidate SjScrib-ligand interactions

The GAL4 AD plasmids expressing the carboxyl-terminus of the candidate ligands of SjScrib-PDZ1 and SjScrib-PDZ4 were first constructed. The self primer template PCR reactions were performed to produce DNA templates encoding the 15 amino acids of extreme carboxyl-terminus of each ligand candidate (The primer pairs were listed in Table S1). The PCR procedure included thirty cycles as follows: 98°C for 5 s; 50°C for 25 s, and 72°C for 1 s, with a final extension at 72°C for 2 min. The resulting DNA fragments were recovered, and further digested with *Eco*R I and *Bam*H I endonucleases, and cloned into the *Eco*R I/*Bam*H I sites of GAL4 AD vector, pGADT7 followed by sequencing. Each constructed plasmid was co-transformed with the corresponding SjScrib-PDZ1 and SjScrib-PDZ4 bait plasmids into yeast strain CG1945, respectively. The positive interactions were selected by Y2H assays as mentioned above.

### Accession numbers

PDB: 1X5Q, PDB: 1WHA, PDB: 1UJU, GenBank: AY809835, GenBank: AY815909, GenBank: XM_002581163, GenBank: AHC92618, GenBank: KF730248, GenBank: CCD76510, GenBank: NP_733154, GenBank: AAL38976, GenBank: XP_002581546, GenBank: FN320595.

## Results and Discussion

### Molecular characteristics of *S. japonicum* Scribble

**Figure 1:**
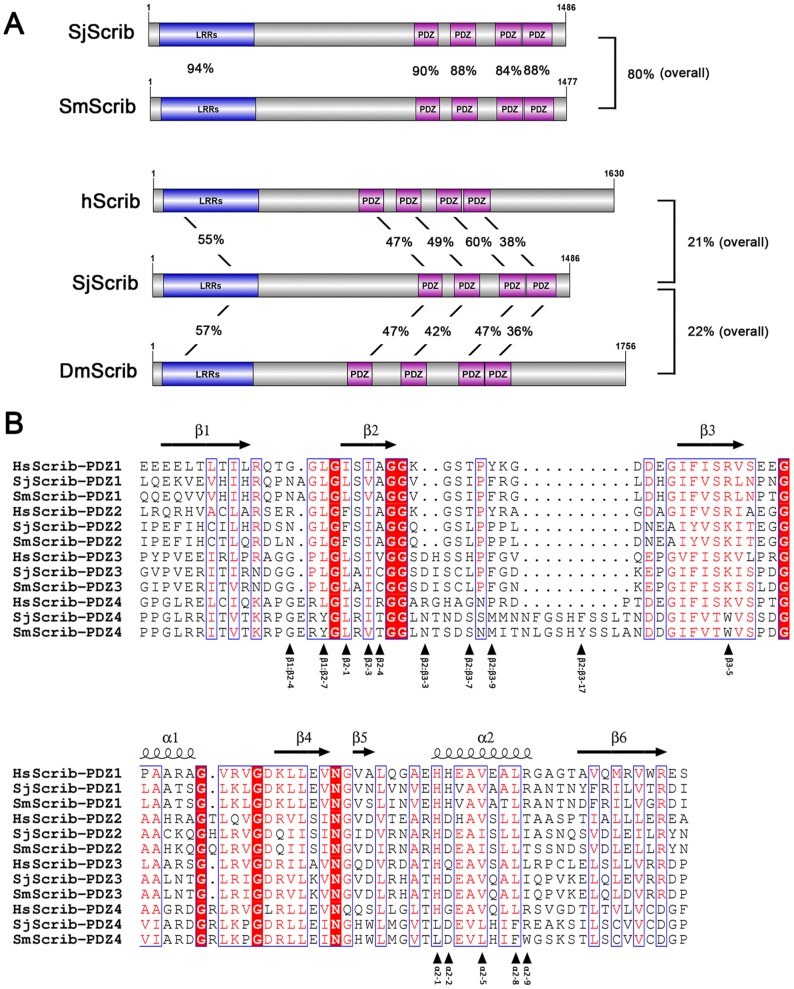
Schematic structures and multi-alignment of PDZ domains of different Scribble orthologs. (A). Diagram of the structures of SjScrib, SmScrib, hScrib, and DmScrib, which consist of an N-terminal leucine-rich repeat (LLR), and four PDZ domains. Sequence identities of full-length protein, as well as LRR and PDZ domains between SjScrib and the orthologs from *S. mansoni*, *H. sapiens*, and *D. melanogaster* are shown between schematic structures. (B). Structure-based sequence alignments of four PDZ domains of Scrib orthologs. Secondary structure elements are indicated above the alignment and refer to the structure of hScrib-PDZ1 domain (PDB code 1X5Q). Each fundamental residue that mediates ligand recognition is denoted and numbered according to its position in an element of secondary structure; and within loops, the residues are numbered according to the sequence of SjScrib-PDZ4. The black arrowheads under the sequence indicate residues that are potentially involved in ligand recognition. Sequence alignment showed that a stretch of 27 amino acids in the second PDZ domain of SmScrib is missing in the sequence of CCD76510 (GenBank accession number). The missing fragment (-AIYVSKITEGGAAHKQGQLRVGDQIIS-) was retrieved from another orthologous sequence (GenBank accession number: XP_002581546).

The *S. japonicum Scribble* gene encodes a protein of 1,486 amino acids, with a theoretical molecular weight of 168 kDa. Homology blast showed that SjScrib shares a relatively high degree of sequence homology with the orthologous *S. mansoni* scribble, SmScrib (GenBank accession number: CCD76510) (Figure 1A). A leucine-rich repeat (LRR) domain within the N-terminus of SjScrib is relatively conserved with orthologs of *Drosophila* and *H. sapiens*, indicating that the function mediated by this domain may be evolutionarily conserved. Four PDZ domains are located in the relatively extreme C-terminal region of schistosome Scribs (Figure 1A). It is obvious that the carboxyl-terminal (CT) domain, which has previously been suggested not being essential for any aspect of Scrib localization or function [17], is missing in SjScrib and SmScrib when compared with DmScrib and hScrib (Figure 1A). Among the four PDZ domains of SjScrib, the third PDZ domain shares the highest degree of homology (60%) with hScrib-PDZ3 (Figure 1A). The peptide sequences of the SjScrib-PDZ domains were aligned with homologous sequences of SmScrib and hScrib (Figure 1B). The secondary structures of the PDZ domains were determined using the NMRs structure of hScrib-PDZ1 (PDB codes 1X5Q) as a template. It is notable that the loop between β2 and β3 in the fourth PDZ domain of schistosome Scribs is longer than that of hScrib-PDZ4, which may increase the flexibility of this domain (Figure 1B). Totally, SjScrib and SmScrib possess the general primary characteristics as that of the homologous proteins of other organisms, indicating that they may play analogous roles in schistosome parasites. The Scribble polarity module is composed of Scribble, lethal giant larvae (Lgl) and discs large (Dlg), which are well conserved across species from worms and flies to mammals [41], [58]. Recently, it has been shown that the *S. japonicum* Lgl protein is localized in the worm tegument and knockdown of this gene can significantly deform the surface structure of adult worm and impair egg hatching [59]. To some extent, these data may reveal the potential function of the Scribble polarity module in *S. japonicum*.

### Homology modeling reveals a different degree of structural divergence between SjScrib-PDZs and hScrib-PDZs

**Figure 2:**
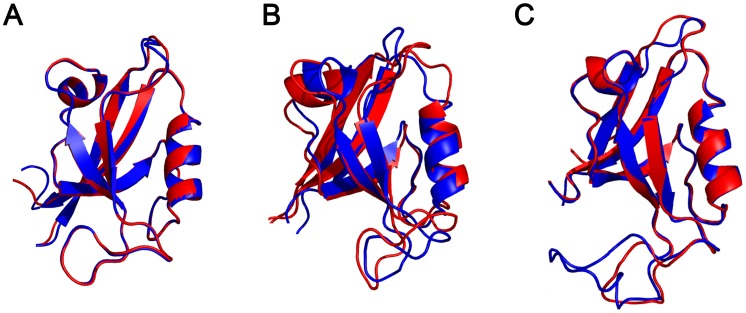
Superimposition of the structures of SjScrib and hScrib PDZ domains. Molecular models of the first (A), second (B), and fourth (C), PDZ domain of SjScrib (blue ribbon) were respectively superimposed on the template structure of the corresponding hScrib-PDZ domain (red ribbon). Superimposition was produced by the program Swiss PDB Viewer [51] and refined by PyMol Viewer program. RMSD (root mean square deviation) value for the first, second, and fourth PDZ domain is 0.41 Å, 1.36 Å, and 0.64 Å, respectively.

To assess the structural divergence between SjScrib and hScrib PDZ domains, we further superimposed the structures of SjScrib-PDZs with three available NMR structures of hScrib PDZ domains. Although the primary sequence identity of the second PDZ domain between SjScrib and hScrib is higher than that of the first and fourth PDZ domains (Figure 1A), the structural similarity of the second PDZ domain between SjScrib and hScrib is the lowest among the three PDZ domains (Figure 2). The root mean square deviation (RMSD) value for the first, second, and fourth PDZ domain is 0.41 Å, 1.36 Å, and 0.64 Å, respectively, indicating that the ligand binding specificity for SjScrib-PDZ2 may be more likely to diverge from that of hScrib-PDZ2 than those of SjScrib-PDZ1 and SjScrib-PDZ4 from the corresponding hScrib PDZ domains, and the ligand binding specificity may be relatively conserved between SjScrib-PDZ1 and hScrib-PDZ1.

### Transcriptional analysis of the *SjScrib* gene at different developmental stages of *S. japonicum*

**Figure 3:**
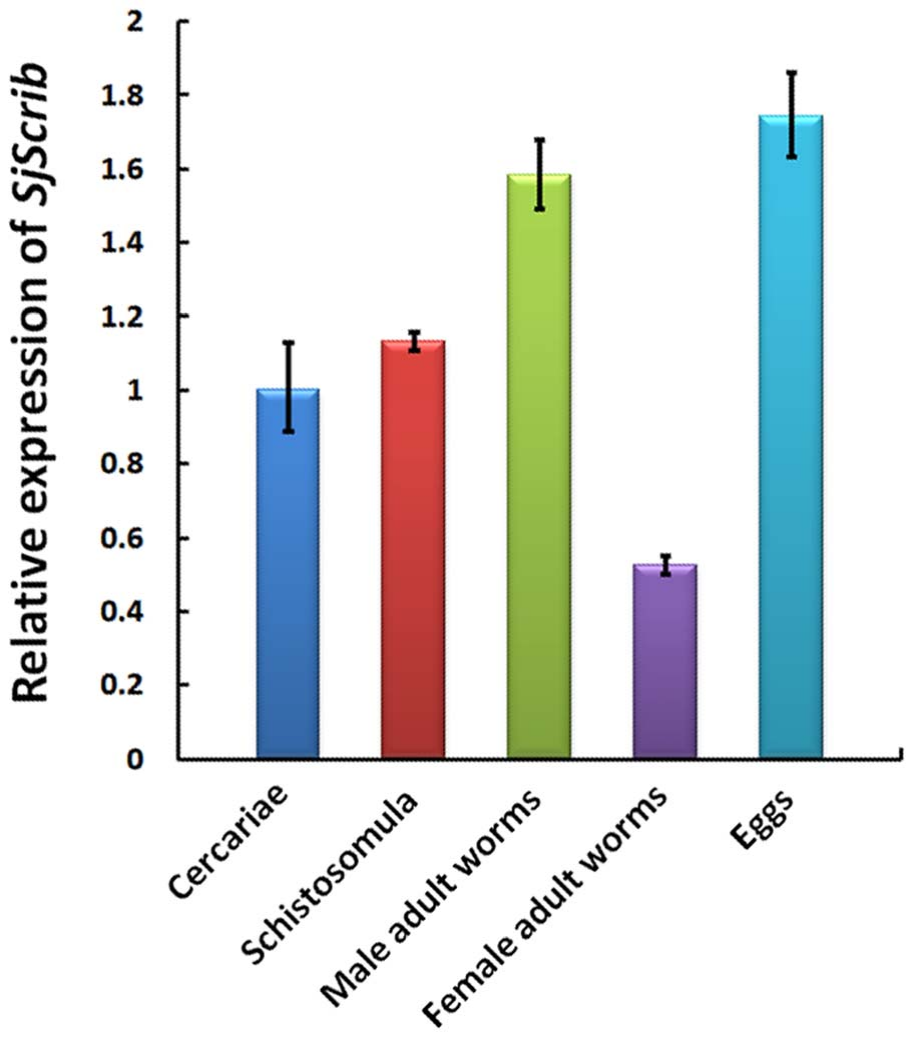
Transcriptional analysis of the *SjScrib* gene at different developmental stages by qRT-PCR. Transcriptional levels were calibrated according to the comparative 2^−ΔΔCt^ method using the housekeeping gene *SjPSMD4* as an endogenous control. The expression of the *SjScrib* gene was normalized relative to the cercarial stage. Error bars represent the standard deviations of the mean from the three replicates.

QRT-PCR was performed to determine the transcriptional profiles of *SjScrib* at different developmental stages and between sexes of the parasite. As a result, we observed that the *SjScrib* gene was ubiquitously expressed during different developmental stages, but in a stage-biased pattern. The transcription levels of the *SjScrib* gene were similar in cercariae and schistosomula at a medium level. In adult worms, the transcriptional level was significantly higher in male than that in female worms. In the egg stage, the expression of *SjScrib* was relatively higher than that in any other stages detected (Figure 3). The data suggest that SjScrib is a necessity for all developmental stages of the parasite, particularly during the development of egg embryo. The reason that *SjScrib* was less transcribed in the female parasites is puzzling, but it could be postulated that in female adult worms, a relatively low number of cells need to maintain membrane polarity when compared to other developmental stages of the parasite.

### Y2H screening against each of SjScrib-PDZ domain

**Figure 4:**
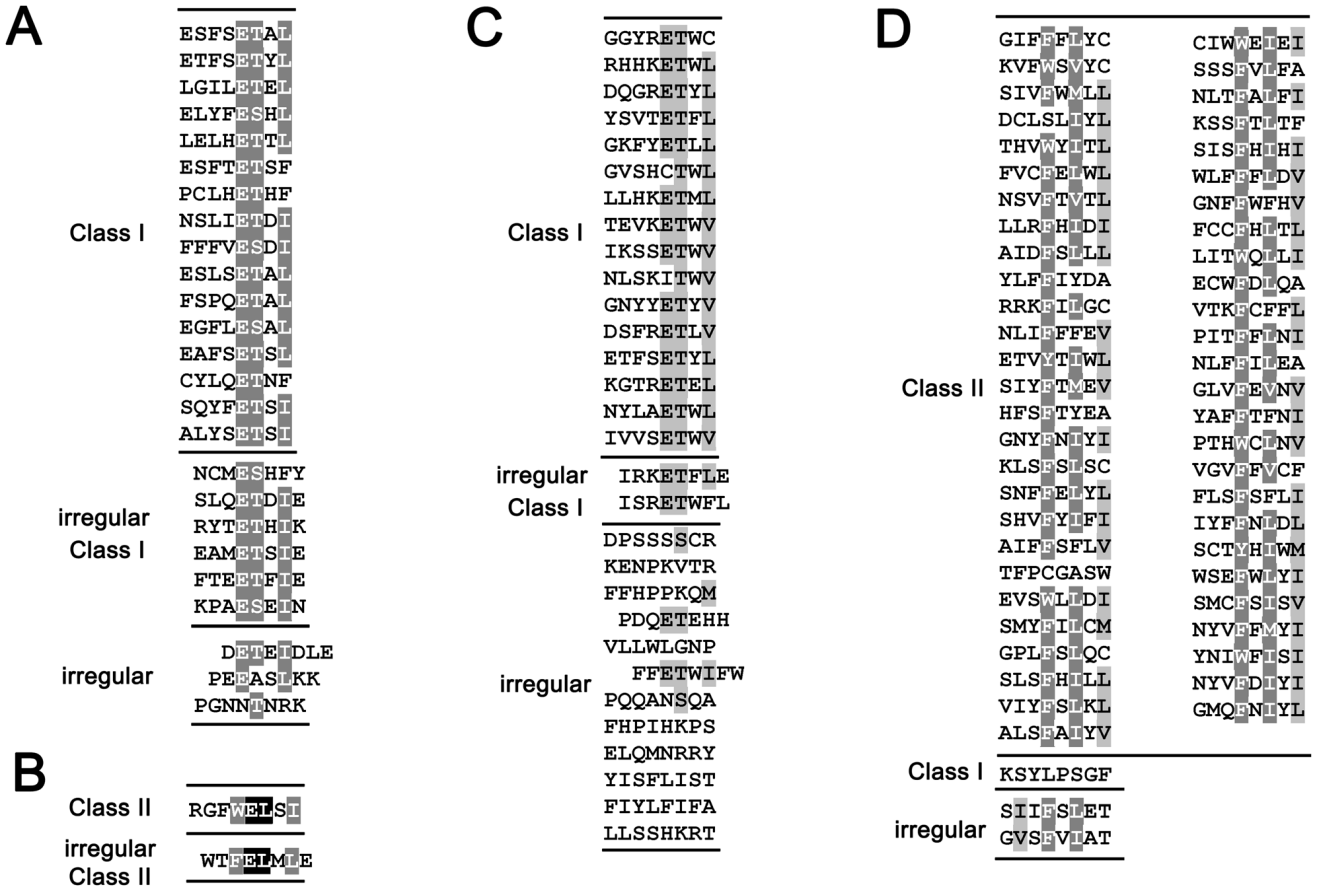
The unique ligand carboxyl termini for each PDZ domain of SjScrib. The unique ligand carboxyl termini for SjScrib-PDZ1 (A), SjScrib-PDZ2 (B), SjScrib-PDZ3 (C), and SjScrib-PDZ4 (D), are shown respectively. The extreme carboxyl termini (8 amino acids) of unique ligands interacted with each SjScrib-PDZ domain were aligned by ClustalX 2.0, respectively, and the alignments were refined with GeneDoc software. Amino acids that are identical or similar are shaded in black and grey, respectively.

To determine the binding properties of SjScrib-PDZs, an arbitrary peptide library was screened by Y2H assays with a similar approach for the determination of the binding specificity of SjGIPC3-PDZ [57]. For SjScrib-PDZ1, a total of 35 positive clones were obtained, which encodes 25 unique carboxy-termini. Among them, 16 belong to Class I PBM, 6 belong to irregular Class I PBM (which can be viewed as a projection of an amino acid at C-terminus of regular PBMs), and 3 cannot be classified (Figure 4A). For SjScrib-PDZ2, only 2 positive clones were probed. One is a Class II PBM, and the other is an irregular Class II PBM (Figure 4B). For SjScrib-PDZ3, 37 positive clones which encode 30 unique carboxy-termini were obtained. Among them, 16 belong to Class I PBM, 2 belong to irregular Class I PBM, and 12 are irregular (Figure 4C). For SjScrib-PDZ4, 58 positive clones encoding 56 unique carboxy-termini were obtained. Among them, 53 belong to Class II PBM, 1 belongs to Class I PBM, and 2 are irregular (Figure 4D).

### Determination of the specific binding sites within irregular ligands

For each PDZ domain of SjScrib, ligands with irregular C-termini were subjected to further analysis. Consequently, putative binding sites were inferred within the internal sequences of these ligands according to the pattern of regular C-terminal PBMs (Table 1, bold and underline characters). To confirm these putative binding motifs, point mutations were introduced into the key amino acid residues and those mutants were further validated in the Y2H system (Table 1).

**Table 1:** Validation the interaction between the mutants and the SjScrib-PDZ domains by Y2H assays.

SjScrib-PDZ4	Ligand sequence and point mutation^1^	Y2H^2^
P1-L8M	CM**ES(A)HF**Y*	×
P1-L10M	SLQ**ET(A)DI**E*	×
P1-L23M	FHSYRYT**ET(A)HI**K*	×
P1-L34M	YPHSEAM**ET(A)SI**E*	×
P1-L35M	SFTE**ET(A)FI**E*	×
P1-L44M	LFLTVTNMVAEKPA**ES(A)EI**N*	×
P1-L1M	SDNHFLHNVTSGCDLIWD**ET(A)EI**DLE*	×
P1-L22M	CDEKYMVVAR**ET(A)NI**EMGFPEEASLKK*	×
P1-L25M	LNIRPKTIKTLEENLGITIQDIGMGKDFMSKTPKAMATKDKIFFKDPTKGFLQ**ET(A)HF**PGNNTNRK*	×
**SjScrib-PDZ2**		
P2-L1M1	SNNVNKMKMVLTKKVTEEEVSINRGLLTPKFEDVPRKNTVTEASVTYAFQRGFWELSI(T)*	×
P2-L1M2	SNNVNKMKMVLTKKVTEEEVSINRGLLTPKFEDVPRKNTVTEASVTYAFQRGFWELS(A)I*	×
P2-L1M3	SNNVNKMKMVLTKKVTEEEVSINRGLLTPKFEDVPRKNTVTEASVTYAFQRGFWEL(T)SI*	×
P2-L1M4	SNNVNKMKMVLTKKVTEEEVSINRGLLTPKFEDVPRKNTVTEASVTYAFQRGFWE(A)LSI*	×
P2-L1M5	SNNVNKMKMVLTKKVTEEEVSINRGLLTPKFEDVPRKNTVTEASVTYAFQRGFW(T)ELSI*	×
P2-L1M6	SNNVNKMKMVLTKKVTEEEVSINRGLLTPKFEDVPRKNTVTEASVTYAFQRGF(T)WELSI*	√
P2-L2M1	CLVTDELWTWTFELML*	√
P2-L2M2	CLVTDELWTWT**FELML(T)**E*	×
P2-L2M3	CLVTDELWTWT**FELM(T)L**E*	×
P2-L2M4	CLVTDELWTWT**FEL(T)ML**E*	×
P2-L2M5	CLVTDELWTWT**FE(A)LML**E*	×
P2-L2M6	CLVTDELWTWT**F(T)ELML**E*	×
**SjScrib-PDZ3**		
P3-L2M	SRKKYIKRWAKDMNRHLSKENIHMPTIRK**ET(A)FL**E*	×
P3-L59M	FHFYPISR**ET(A)WF**L*	×
P3-L3M	SFQSSKCRVIFKETNIKAEV**TT(A)VC**VYVCLMSEKEPIDSKRPEFPSLIPPGWASIRDPSSSSCR*	×
P3-L34M	GN**YT(A)SI**IFFPGKKENPKVTR*	×
P3-L35M	SSAAGTKHNV**ST(A)II**FFHPPKQM*	×
P3-L41M	NF**TT(A)VI**LLHCQHPDQETEHH*	×
P3-L50M	SPGFCLRPSRKCPCGSSK**TT(A)VL**LWLGNP*	×
P3-L51M	SSTLNLVKPHFF**ET(A)WI**FW*	×
P3-L56M	WLNSPRLSIGPFTMQNI**TT(A)VI**YFRGFTNSNSVKFSCLSLLSSWDYRHAPPQQANSQA*	×
P3-L70M	LRQPEHYWAWGLSPYEYLECLPKDRQKKAQTVR**TT(A)II**LFHPIHKPS*	×
P3-L79M	NFKVFSPLDREKVWF**ST(A)CI**LFRRSSEYESSAVNEKIPKLNSTRVGIDTGSIELELQMNRRY*	×
P3-L104M	FIR**ET(A)WV**QKSKAVSYISFLIST*	×
P3-L117M	NS**ST(A)FI**YLFIFA*	×
P3-L134M	FPLKHFTVLHTQ**ST(A)CV**LFLLSSHKRT*	×
**SjScrib-PDZ4**		
P4-L4M1	LRKITRPHAINNHRPEKEKTYFQYKKELVKSGRGWESSG**WQLCA(T)**DCLSLIYL*	×
P4-L4M2	LRKITRPHAINNHRPEKEKTYFQYKKELVKSGRGWESSG**WQL(T)CA**DCLSLIYL*	×
P4-L4M3	LRKITRPHAINNHRPEKEKTYFQYKKELVKSGRGWESSG**W(T)QLCA**DCLSLIYL*	×
P4-L32M1	ASALIRYGRKILNSCWCLFRSSFLPGSHECRGVDIHKLWEGKVRNARFYLARPSFVTTFPCGASW(T)*	×
P4-L32M2	ASALIRYGRKILNSCWCLFRSSFLPGSHECRGVDIHKLWEGKVRNARFYLARPSFVTTFPCGA(T)SW*	×
P4-L32M3	ASALIRYGRKILNSCWCLFRSSFLPGSHECRGVDIHKLWEGKVRNARFYLARPSFVTTF(T)PCGASW*	×
P4-L39M1	YSPLPLMARRYKSYLPSGF(T)*	×
P4-L39M2	YSPLPLMARRYKSYLPS(R)GF*	×
P4-L39M3	YSPLPLMARRYKSY(T)LPSGF*	×
P4-L8M1	SIIFSLET(R)*	×
P4-L8M2	SIIFSL(T)ET*	×
P4-L8M3	SIIF(T)SLET*	×
P4-L42M1	CFIMCFVIRKRNGVSFVIAT(R)*	×
P4-L42M2	CFIMCFVIRKRNGVSFVI(T)AT*	×
P4-L42M3	CFIMCFVIRKRNGVSF(T)VIAT*	×

1 : The potential PBMs within the irregular ligands of each PDZ domain of SjScrib are shown in bold and underline. The substituted residues are indicated in brackets.

2 : √: positive in the Y2H system; ×: negative in the Y2H system.

For SjScrib-PDZ1, a core binding sequence was deduced from its canonical C-terminal PBMs, -[E][TS][x][LIF]* (Figure 4A). A putative binding motif, [E][TS][x][IF], was inferred for each irregular ligand. Substitution of the hydrophobic residue Ala for the polar residue Thr/Ser totally interrupted the interactions between SjScrib-PDZ1 and the irregular ligands. For SjScrib-PDZ2, only two unique sequences were obtained, probably due to the stringent recognition at particular binding sites. We hypothesized that the typical II C-terminal PBM, -WELSI* and the atypical II C-terminal PBM, -FELMLE* with one amino acid Glu projected at the C-terminus of the binding site, are likely to be the core binding motifs for SjScrib-PDZ2. This hypothesis was confirmed by a series of point mutations introduced into each amino acid of the putative PBMs (Table 1). For SjScrib-PDZ3, a putative core binding sequence, -[E][T][*Φ*][*Φ*] was deduced based on its canonical typical I C-terminal PBMs (Figure 4C). Potential PBM was postulated for each irregular ligand of SjScrib-PDZ3. Substitutions the conserved polar residue Thr with the hydrophobic residue Ala led to the failure of the interactions between the PDZ domain and these mutants (Table 1). For SjScrib-PDZ4, a consensus sequence -[F_WY_][x][*Φ*][x][*Φ*]* was inferred from most of the typical C-terminal PBMs (Figure 4D). The binding sites within the five irregular ligands were located based on the consensus sequence. Two of them, P4-L32 and P4-L39 were found to be particularly unusual, as they potentially bear 7 and 6 amino acids, respectively, for interacting with SjScrib-PDZ4. However, as residues of small size are dominant within both PBMs (“PCGAS” in P4-L32 and “PSG” in P4-L39), it is reasonable to speculate that they can be well accommodated within the binding groove of SjScrib-PDZ4. To confirm our prediction, three point mutations were introduced in each of the five putative binding sites (Table 1), and as a result, these mutants were found to be unable to bind to SjScrib-PDZ4. Together, these data support our speculation that SjScrib-PDZ1, SjScrib-PDZ3, and SjScrib-PDZ4 have the ability to bind both C-terminal and internal PBMs, which will be further discussed below.

### Determination of ligand binding specificities of the SjScrib-PDZ domains based on the statistical analysis of amino acid composition within C-terminal and internal PBMs

**Figure 5:**
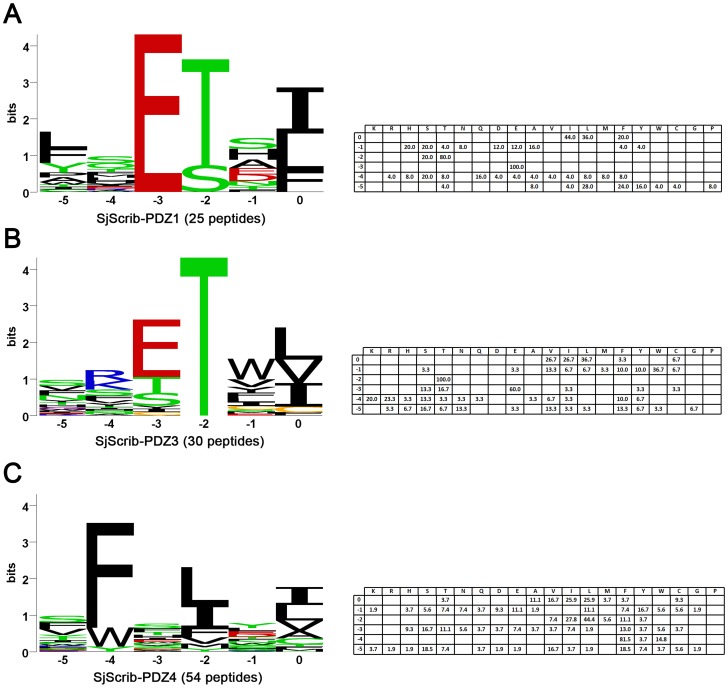
Ligand binding specificities of SjScrib-PDZ1, SjScrib-PDZ3, and SjScrib-PDZ4. Each aligned C-terminal and internal PBMs set was used to create a position weight matrix (PWM) for SjScrib-PDZ1 (A), SjScrib-PDZ3 (B), SjScrib-PDZ4 (C), respectively. Two irregular PBMs (-TFPCGASW* and -KSYLPSGF*) were excluded from the analysis of the ligand binding specificity of SjScrib-PDZ4. The percentages of each type of the amino acids at a particular position (from P^−5^ to P^0^) are presented in the tables on the right panel.

SjScrib-PDZ1 showed particular preference for hydrophobic residues at the carboxyl terminus of its ligand binding site (P^0^, not the extreme carboxyl terminus of the ligand), especially Ile (44.0%), Leu (36.0%), or Phe (20.0%). At P^−1^, no clear preference was observed. At P^−2^, only the polar amino acids Thr (80.0%) and Ser (20.0%) were selected. At P^−3^, Glu (100%) was absolutely preferred. At P^−5^, the hydrophobic residues, such as Leu (28.0%), Phe (24.0%) and Tyr (16.0%), along with a rare occurrence of other hydrophobic residues were selected (Figure 5A). Based on the statistical analysis of its unique PBMs, a consensus-binding sequence was determined as -[*Φ*][x][E][TS][x][ILF] for SjScrib-PDZ1.

Hydrophobic residues, especially Leu (36.7%), Ile (26.7%), or Val (26.7%), were also predominantly preferred at P^0^ site of SjScrib-PDZ3 ligands. At P^−1^, the preference for Trp (36.7%) was observed, followed by other hydrophobic residues Val (13.3%), Phe (10.0%) and Tyr (10.0%). The P^−2^ site demonstrated an absolute preference for the polar residue Thr (100%). At P^−3^, Glu (60.0%) was predominantly selected, along with a rare selection of residues Thr (16.7%) and Ser (13.3%). While the positive charged residues, Arg (23.3%) and Lys (20.0%) were slightly prone to be at P^−4^ site. At P^−5^ site, no obvious preference was observed (Figure 5B). Based on these results, a consensus-binding sequence, -[x][RKx][E_TS_][T][W*Φ*][ILV], was deduced for SjScrib-PDZ3.

For the ligands of SjScrib-PDZ4, hydrophobic residues, especially, Ile (25.9%), Leu (25.9%), or Val (16.47%), were preferred at P^0^, with a weak preference for the residues of Ala (11.1%) and Cys (9.3%). The P^−2^ site was also occupied by the hydrophobic residues of Ile (44.4%) and Leu (27.8%). At P^−4^, Phe (81.5%) was predominantly selected, with a less occurrence of Trp (14.8%). No clear preference was observed for the sites of P^−1^, P^−3^ and P^−6^ (Figure 5C). Therefore, a consensus-binding sequence, -[x][F_W_][x][IL][x][ILV], was inferred for SjScrib-PDZ4 based the amino acid occurrence at particular positions.

### Ligand specificity analysis by comparison of SjScrib-PDZs and other LAP family PDZ domains according to the key residues for ligand recognition

**Figure 6:**
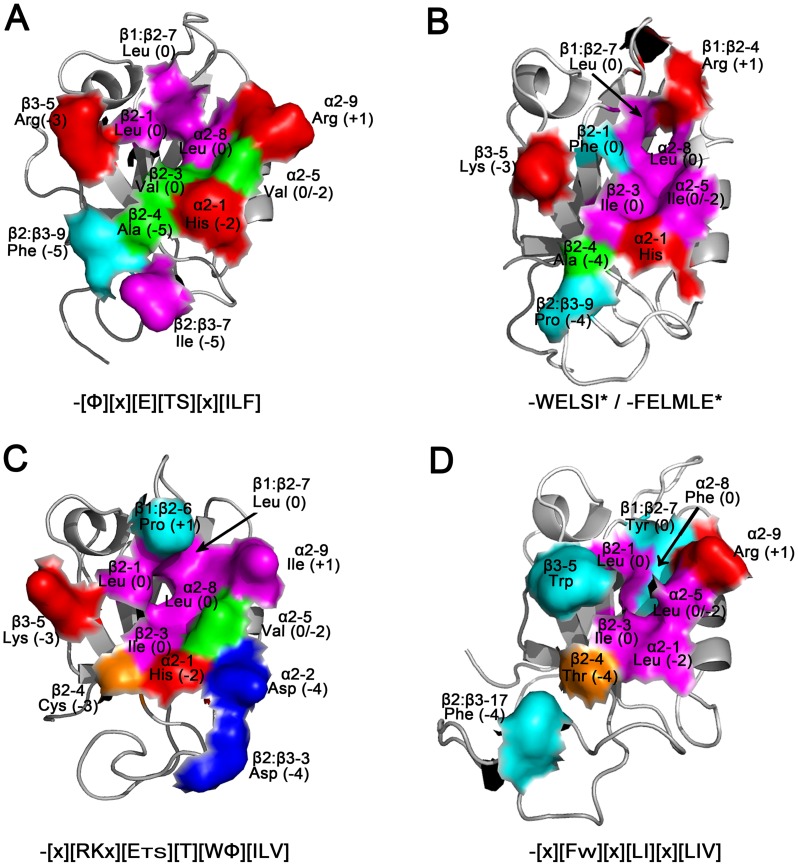
Modeling of ligand binding specificities for the SjScrib-PDZ domains. The predicted structures of the first (A), second (B), third (C), and fourth (D) PDZ domain of SjScrib are shown and colored to highlight the functional residues (shown as a partial molecular surface model) involved in ligand recognition. The position in an element of secondary, name, and the potential ligand subsite involved in recognition for each key residue are denoted. Color schemes: large hydrophobic residues (magenta); small hydrophobic residues (green); small polar residues (orange); aromatic residues (cyan); positively charged residues (red); negatively charged residues (blue). The ligand specificities for each PDZ domain are shown under each panel. Structural figures were produced with PyMOL.

The comparative structural analysis of human LAP family members ZO-1-PDZ1 and Erbin-PDZ has provided a valuable insight for ligand recognition across the entire binding site [60]. To better understand the molecular basis for ligand recognition of SjScrib-PDZ domains, comparative analyses were conducted for ligand specificities of the SjScrib-PDZ domains according to the key residues for ligand recognition. It has been suggested that the hydrophobic residue at β2-1 is a key determinant of the P^0^ specificity of ZO-1-PDZ1 and Erbin-PDZ. The PDZ domains that show a stringent preference for a Val^0^ side chain contain a Phe at position β2-1, whereas those that can accommodate a larger Leu/Ile at site 0 contain a Leu/Ile at the same position [60]. Here, however, it seems that SjScrib-PDZ1, SjScrib-PDZ3 and SjScrib-PDZ4, but not SjScrib-PDZ2, complies with this rule, suggesting that other residues within the hydrophobic pocket (i.e. β2-3, α2-5, and α2-8) may also affect the P^0^ specificity of LAP family PDZ domains (Figure 6) [60].

The PDZ domains of LAP family that contain His and Val at α2-1 and α2-5, respectively, usually have a strong preference for Thr/Ser at P^−2^ [60]. Strikingly, SjScrib-PDZs obey this rule quite well, i.e., SjScrib-PDZ1 and SjScrib-PDZ3 that bear His and Val at α2-1 and α2-5, respectively prefer Thr at P^−2^ (Figure 6A and C), whereas SjScrib-PDZ2 that has an Ile at α2-5 and SjScrib-PDZ4 that has a Leu at α2-1 prefer Ile and Leu at P^−2^, respectively (Figure 6B and D). Also, it has been suggested that the positively charged residue Arg or Lys at position β3-5 of the LAP family PDZ domains favors the electrostatic interactions with a negatively charged ligand side chain at P^−3^ [60]. A similar situation was observed in regarding of the selectivity of P^−3^ of SjScrib-PDZs. SjScrib-PDZ1, SjScrib-PDZ2, and SjScrib-PDZ3 that contain an Arg or a Lys at position β3-5, exhibit a strong preference for a Glu at P^−3^ (Figure 6A, B, and C). In contrast, SjScrib-PDZ4 that has a Trp at position β3-5 displays promiscuity for residues at P^−3^ (Figure 6D).

In addition, SjScrib-PDZ2 and SjScrib-PDZ4 display a preference for ligands that contain an aromatic side chain at P^−4^. Both recognitions are predominantly mediated by residues within the β2:β3 loop. In the case of SjScrib-PDZ2, the side chain of Phe/Trp^−4^ may interact with residues in β2 (Ala (β2-4)) and the β2:β3 loop (Pro (β2:β3-9) (Figure 6B). While in the case of SjScrib-PDZ4, the aromatic side chain at site -4 of its PBMs may potentially interact with Thr (β2-4) and Phe (β2:β3-17), based on the structural analysis (Figure 6D). However, the interactions between the PDZ domains and the artificial ligands may not completely reflect the nature of the molecular recognitions *in vivo*, thus approaches such as co-immunoprecipitation with native SjScrib will be needed to further address the binding capacity of these domains.

### Divergence of ligand specificities of SjScrib-PDZs and hScrib-PDZs

Zhang *et al.* have previously investigated the binding specificity profiles of a panel of PDZ domains of LAP proteins by phage display screening [43]. They found that all four C-terminal residues of the ligands constituted a core recognition motif for interactions, while the more upstream residues also supported the core binding sites. Here, we performed a comparative analysis to figure out the convergent and divergent ligand specificities between SjScrib-PDZs and hScrib-PDZs. The ligand binding profile for SjScrib-PDZ1 (-[*Φ*][x][E][TS][x][ILF]) was found to be similar with that of hScrib-PDZ1 (-[*Φ*][*Φ*][E][TS][x][VIL]*), but not exactly the same. For example, hScrib-PDZ1 prefers a hydrophobic residue at P^-4^, whereas this is not the case for SjScrib-PDZ1. Except that both PDZ domains prefer the hydrophobic residues Leu and Ile at P^0^, SjScrib-PDZ1 exhibits a preference of the aromatic residue Phe; whereas hScrib-PDZ1 prefers an aliphatic residue Val at this site. The binding specificity pattern for SjScrib-PDZ3 (-[x][RKx][E_TS_][T][W*Φ*][ILV]) resembles that of hScrib-PDZ3 (-[*Φ*][*Φ*][EDQH][TS][x][LIV]*) [43], but with certain distinctions for SjScrib-PDZ3. For instance, SjScrib-PDZ3 shows no clear preference of hydrophobic residues at P^−5^. At P^−4^, unlike hScrib-PDZ3 that prefers hydrophobic residues, SjScrib-PDZ3 exhibits a preference for the positively charged residues, Arg and Lys, which may form electrostatic interactions with the negatively charged residue Asp at α2-2 or β2:β3-3 (Figure 6C). Further, there is a strong preference for hydrophobic residues, particularly the aromatic residue Try, at P^−1^ for SjScrib-PDZ3, which was not the case for hScrib-PDZ3.

The most significant divergence was observed when comparing the binding preferences of SjScrib-PDZ2 with that of hScrib-PDZ2. In contrast to hScrib-PDZ2, which prefers typical I C-terminal PBMs, -[*Φ*][*Φ*][ED][TS][W][V]* [43], SjScrib-PDZ2 displays a preference for a typical II PBM, -WELSI*, and an irregular typical II PBM, -FELMLE*. Also, SjScrib-PDZ2 exhibits no preference of hydrophobic residues and Tyr at P^−5^ and P^−1^, respectively, in contrast to that observed in those of hScrib-PDZ2. These results are consistent with that of homology modeling, i.e. the structural similarity between SjScrib-PDZ2 and hScrib-PDZ2 is poor (Figure 2B). As no binding preference of hScrib-PDZ4 is available so far, we cannot directly compare the binding specificity profile of SjScrib-PDZ4 with that of hScrib-PDZ4. However, given the relatively low homology between SjScrib-PDZ4 and hScrib-PDZ4 (Figure 1A), as well as the difference in several key residues involved in ligand recognition, such as β3-5, α2-1, β1:β2-7, β2:β3-7, β2-4, and α2-8 between the two domains (Figure 1B) [60], we postulate that the binding specificity of SjScrib-PDZ4 may be highly divergent from that of hScrib-PDZ4. Totally, these different ligand binding characteristics between the homologous PDZ domains of SjScrib and hScrib can be potentially utilized for the drug design against SjScrib.

### The internal ligand binding ability of SjScrib-PDZs

For the first time, the internal motifs binding ability of the three SjScrib-PDZ domains, SjScrib-PDZ1, SjScrib-PDZ3, and SjScrib-PDZ4 was confirmed in this study. Based on the ratio of internal PBMs to C-terminal PBMs obtained from each SjScrib-PDZ, we can infer that SjScrib-PDZ3 (12∶18) displays a relatively strong preference for internal motif binding, followed by SjScrib-PDZ1 (3∶22); while SjScrib-PDZ4 (1∶55) shows a weak ability of internal motif recognition. Interestingly, preferences at P^−3^ between the C-terminal and internal PBMs of SjScrib-PDZ3 are different. The C-terminal PBMs of SjScrib-PDZ3 display a strong preference for Glu^−3^; whereas its internal PBMs show a preference for Thr/Ser^−3^, which may interact with the residue Cys at β2-4 (Figure 6C) as previously suggested [60]. Also, the negatively charged residues (Glu and Asp) frequently appear at the position +1 (P^+1^) (the residue beyond the core binding motif) in these irregular and internal PBMs of SjScrib-PDZ1 (Table 1). In contrast, the hydrophobic residues (Leu, Ile, and Val) or the aromatic amino acids (Phe and Tyr) are preferred at this position in the irregular and internal PBMs of SjScrib-PDZ3. The structural analysis indicated that the P^+1^ residue may interact with the residues of the carboxylate-binding loop or α2-9 (Figure 6). In the case of SjScrib-PDZ1, the Glu/Asp^+1^ may form electrostatic interactions with the positively charged residue Arg at position α2-9 (Figure 6A), while the hydrophobic residues at P^+1^ of the irregular PBMs of SjScrib-PDZ3 potentially interact with Pro (β1:β2-6) and Ile (α2-9) (Figure 6C). Similar to the case of SjScrib-PDZ1, the Glu^+1^ of the atypical PBM of SjScrib-PDZ2 (P2-L2) and the Asp^+1^ in the internal ligand of SjScrib-PDZ4 (P4-L4) can potentially interact with Arg (β1:β2-4) (Figure 6B) and Arg (α2-9) (Figure 6D), respectively. These results indicate that SjScrib-PDZs may favor the unconventional interactions with a mode of Par6-Pals internal recognition [61].

In *Drosophila* and zebra fish, it has been shown that some PDZ domains of Scribble can interact with ligands via internal recognition [24], [62]. These data together with ours suggest that the internal interaction pattern of the Scrib-PDZ domains may be more common than currently appreciated. The internal binding model of PDZ domains can dramatically increase the diversity of its native ligands. Thus, it is possible that SjScrib may mediate multiple biological pathways through a manner of internal recognition. So far, several compounds screened based on the internal binding specificities of PDZ domains have shown their therapeutic potential against several diseases with less side effects [11]. The finding of the internal binding preferences of SjScrib-PDZs has raised an opportunity for designing small molecular drugs against this target.

### Potential native ligand prediction and validation for the SjScrib-PDZ domains

To identify the native ligand candidates of SjScrib-PDZs, the Tailfit program was employed to search potential ligands in the *S. japonicum* predicted proteomic database (sjr2_protein.fasta) with less stringent core binding motifs as baits (Table 2). A panel of protein sequences was retrieved. After filtering, 2 and 9 proteins were selected as potential ligand candidates for SjScrib-PDZ1 and SjScrib-PDZ4, respectively (Table 2). However, only one protein, Prepro-cathepsin C, was confirmed to be potential ligand of SjScrib-PDZ4 in the Y2H system. Although the C-terminal tails of most of the candidates fit the ligand consensus sequence quite well, they are not native partners of SjScrib. One possibility is that the interaction between PDZ domain and ligand partner was not simply determined by the consensus sequence. For example, the consensus sequence ([x][F_W_][x][IL][x][ILV]) of SjScrib-PDZ4 is just a “primary”, but not “tertiary” determinant of specificity. The characteristics, such as the charge and size of the residues at position -1 and -3, may also affect the ligand recognition. These residues must coordinate with the ones at position 0, -2 and -4 and function as a whole determinant for recognition. Vice verse, this fact may explain why some unusual PBMs (e.g. P4-L32, -FPCGASW* and P4-L39, -YLPSGF*) could interact with SjScrib-PDZ4, although they do not fit the typical consensus sequence well. As it has been suggested that each PDZ domain is capable of interacting with 17 partners on average [63], it is obvious that more potential native ligands of SjScrib are still under discovery, which probably due to the poor quality of the proteomic database, or in the other case, these PDZ domains may interact with the native ligands via internal recognition. Also, it is worth noting that this positive ligand confirmed in Y2H assay is merely a potential partner of SjScrib at current stage, since we expressed the two proteins (the PDZ domain and the tail of Cathepsin C), which may be from different tissues of the parasite under native condition, into one yeast cell. Co-localization assay of the two proteins in different developmental stages of the parasite may further address whether this interaction indeed takes place *in vivo*.

**Table 2:** Validation of the predicted ligand candidates of SjScrib-PDZ1 and SjScrib-PDZ4 in the Y2H system.

Domain	Bait	GenBank Accession No.	Code	C-terminus (*S. japonicum*)	Protein Description	Y2H^#^
SjScrib-PDZ1	[E][TS][x][LIF]*	AAX25037	SP1-1	RDWLSPETEL*	hypothetical protein	×
		CAX73718	SP1-2	SRFVNFETPF*	Lysosomal protective protein precursor	×
SjScrib-PDZ4	[F][x][LIFV][x][LIVA]*	CAX72171	SP4-1	ASNASFPLLL*	Cathepsin L	×
		AAW27318	SP4-2	TKDDAFILSI*	hypothetical protein	×
		CAX72944	SP4-3	NSIDLFSLPA*	putative TatD DNase domain containing 1	×
		AAX30137	SP4-4	DLGPDFKIIL*	hypothetical protein	×
		AAP06275	SP4-5	LLMYIFTLVV*	putative phosphate transporter	×
		CAX75377	SP4-6	FLTSAFAVII*	Sorcin	×
		AAW25664	SP4-7	EDYIRFTVAI*	similar to calpain	×
		AAC32040	SP4-8	SIAIKFDVVL*	prepro-cathepsin C	√
		AAW25594	SP4-9	FGNFLFYIYL*	putative zinc finger protein	×

#: √: positive in the Y2H system; ×: negative in the Y2H system.

In summary, for the first time, we presented the molecular characterization of the PDZ domain-containing protein Scribble from *S. japonicum*. We defined the ligand binding specificities of the SjScrib-PDZ domains by screening a random peptide library in the Y2H system. The convergent and divergent ligand specificities between the SjScrib-PDZ domains and those of its human ortholog were denoted. The confirmation of internal ligand specificities and the identification of irregular ligands for the SjScrib-PDZ domains will assist in the rational design of novel drugs against the parasite. The strategy used here can also be generically applied to the determination of the ligand binding specificities of other parasite-derived PDZ domains.

## Supporting Information

Table S1 **Primer sequences used in this study.**
(XLS)
